# Cardiovascular magnetic resonance feature tracking strain analysis for discrimination between hypertensive heart disease and hypertrophic cardiomyopathy

**DOI:** 10.1371/journal.pone.0221061

**Published:** 2019-08-21

**Authors:** Ulf Neisius, Lana Myerson, Ahmed S. Fahmy, Shiro Nakamori, Hossam El-Rewaidy, Gargi Joshi, Chong Duan, Warren J. Manning, Reza Nezafat

**Affiliations:** 1 Department of Medicine (Cardiovascular Division), Beth Israel Deaconess Medical Center and Harvard Medical School, Boston, MA, United States of America; 2 Department of Radiology, Beth Israel Deaconess Medical Center and Harvard Medical School, Boston, MA, United States of America; Faculty of Medical Science - State University of Campinas, BRAZIL

## Abstract

**Background:**

Hypertensive heart disease (HHD) and hypertrophic cardiomyopathy (HCM) are both associated with an increased left ventricular (LV) wall thickness. Whilst LV ejection fraction is frequently normal in both, LV strain assessment could differentiate between the diseases. We sought to establish if cardiovascular magnetic resonance myocardial feature tracking (CMR-FT), an emerging method allowing accurate assessment of myocardial deformation, differentiates between both diseases. Additionally, CMR assessment of fibrosis and LV hypertrophy allowed association analyses and comparison of diagnostic capacities.

**Methods:**

Two-hundred twenty-four consecutive subjects (53 HHD, 107 HCM, and 64 controls) underwent 1.5T CMR including native myocardial T_1_ mapping and late gadolinium enhancement (LGE). Global longitudinal strain (GLS) was assessed by CMR-FT (CVi42, Circle Cardiovascular Imaging Inc.).

**Results:**

GLS was significantly higher in HCM patients (-14.7±3.8 vs. -16.5±3.3% [HHD], P = 0.004; or vs. -17.2±2.0% [controls], P<0.001). GLS was associated with LV mass index (HHD, R = 0.419, P = 0.002; HCM, R = 0.429, P<0.001), and LV ejection fraction (HHD, R = -0.493, P = 0.002; HCM, R = -0.329, P<0.001). In HCM patients, GLS was also associated with global native T_1_ (R = 0.282, P = 0.003), and LGE volume (ρ = 0.380, P<0.001). Discrimination between HHD and HCM by GLS (c = 0.639, 95% confidence interval [CI] 0.550–0.729) was similar to LV mass index (c = 0.643, 95% CI 0.556–0.731), global myocardial native T_1_ (c = 0.718, 95% CI 0.638–0.799), and LGE volume (c = 0.680, 95% CI 0.585–0.775).

**Conclusion:**

CMR-FT GLS differentiates between HHD and HCM. In HCM patients GLS is associated with myocardial fibrosis. The discriminatory capacity of CMR-FT GLS is similar to LV hypertrophy and fibrosis imaging markers.

## Introduction

Considering an aging population with an increased prevalence of hypertension, the differential diagnosis for patients with increased left ventricular (LV) wall thickness (LVWT) represents a diagnostic challenge often encountered in cardiovascular imaging laboratories. Next to hypertensive heart disease (HHD) [1], hypertrophic cardiomyopathy (HCM) with a frequency of 1/500 in the general population [2,3] shares the phenotype.

Until late disease stages, HHD and HCM exhibit normal systolic function by conventional measurements, such as LV ejection fraction or fractional shortening [2–4]. Yet an early decline in myocardial mechanics measured by strain analysis has been observed in both [5–13]. For instance, longitudinal strain by speckle tracking echocardiography (STE) differentiates hypertensive patients with normal cardiac function and dimensions [8] as well as phenotype negative genotype positive HCM [14] cases from controls.

In hypertensive rats, diffuse subendocardial fibrosis correlates with reduced longitudinal contractility, whilst midwall fibrosis occurs only in later disease stages and correlates with a decline in circumferential strain [15]. Correspondingly, clinical observations showed a decline in longitudinal strain in non-hypertrophied hypertensive patients [8], whilst circumferential strain is only significantly reduced in hypertensive patients with left ventricular hypertrophy (LVH) [5,8]. In HCM patients, strain attenuation is more related to myocyte disarray [16] rather than fibrosis. Since myocyte disarray is widely distributed including areas of normal wall thickness and typically involves >20% of the myocardium [17], it might impact more significantly on strain measurements than pathophysiological processes in HHD.

On histology, HHD and HCM patients share myocyte hypertrophy and fibrosis [18,19]. The latter can be divided into replacement and diffuse fibrosis, which are quantifiable by cardiovascular magnetic resonance (CMR) imaging through late gadolinium enhancement (LGE) [20] and native myocardial T_1_ mapping [21], respectively. Both have been reported to be more prominent in HCM [22] and to be associated with a decline of regional strain measurement [23].

Recently, CMR myocardial feature tracking (CMR-FT), a software solution similar to STE [24], has been introduced for high-resolution evaluation of global and regional myocardial mechanics. CMR-FT tracks the epi- and endocardial borders and has been evaluated in a wide range of cardiovascular disease [24]. Related to the excellent image quality of CMR, CMR-FT has an excellent intra- and interobserver agreement for longitudinal strain [25], as well as good agreement with STE [26].

Considering the different pathophysiological processes in HHD and HCM and their related cardiac mechanics, we hypothesized that CMR-FT global longitudinal strain (GLS) would differentiate between diseases. We also postulated that CMR-FT strain would be associated with myocardial fibrosis and hypertrophy measurements.

## Material and methods

The study was approved by the Beth Israel Deaconess Medical Center’s Institutional Review Board (Protocol Number: 2001P-000793). Written consent was obtained. Two hundred and twenty-four consecutive subjects, referred for CMR at our center between July 2014 and March 2018 and meeting the criteria described below, were included in this retrospective study. The same dataset was previously used for radiomic analysis of myocardial native T_1_ imaging to differentiate between HHD and HCM and was reported in [27]. In the current study we however address a different hypothesis: GLS computed by CMR-FT can discriminate between both diseases. The investigated patients groups (healthy controls, HHD and HCM patients) were defined based on established diagnostic criteria and related CMR measurements [2,3,28–30].

Patients with HCM (n = 107) demonstrated either normal LV cavity size with maximum LVWT ≥15 mm, or LVWT above the normal range (≥12 mm) [30] in the context of high clinical suspicion (i.e. apical variant phenotype, HCM family history + LVWT ≥13 mm), both not explained by loading conditions [2,3]. HHD (n = 53) was defined as increased LVWT (≥12 mm) in the presence of arterial hypertension [28] (i.e. systolic blood pressure ≥140 mmHg on two separate occasions, documented diagnosis of hypertension) and absence of LV cavity dilatation, severe chronic kidney disease, and cardiac disease associated with a similar magnitude of hypertrophy. All control subjects (n = 64) had normal cardiac dimensions/volumes, normal cardiac function, and absence of LGE in common and lacked a history of cardiac disease. Subjects were excluded from the study based on an established diagnosis of Anderson-Fabry disease, amyloidosis, or iron-deposition, history of ST-segment elevation myocardial infarction, evidence of inflammatory processes in the myo- or pericardium, and athletic activity with sufficient duration, intensity and frequency to explain abnormal LVWT.

### CMR imaging and analysis

CMR images were acquired with a 1.5T scanner (Achieva, Philips Healthcare, Best, The Netherlands) equipped with a 32-element cardiac coil. Breath-hold, retrospectively electrocardiogram (ECG)-gated cine, balanced steady state free-precision (bSSFP) images were recorded in the LV 2- and 4-chamber long-axis views, and a short-axis stack covering the entire LV (8-mm slices with 2-mm gaps).

CMR images were interpreted using ViewForm software (Release 4, Philips Healthcare). LV and right ventricular volumes were quantified by manually tracing the end-diastolic and end-systolic endocardial contours and applying a summation of discs method. Slices at the base of LV were included if >50% of the blood pool was encircled by myocardium. LV dimensions or the maximum LVWT were quantified in the short-axis view at the level of the chordae or at the slice with largest endo- to epicardial border distance, respectively. LV mass was calculated as the total myocardial volume without papillary muscles multiplied by the specific gravity of myocardial tissue (1.05 g/mL). LV mass index (LVMI) ≥81 g/m^2^ in men and ≥62 g/m^2^ in women [29] were used to defined LVH. LV end-diastolic volume index >105 ml for men and >96 ml for women [31] were used to defined LV cavity dilatation. Asymmetric or apical hypertrophy were defined as maximum LVWT ≥12 mm [30] with a septal to posterior free wall ratio (interventricular septum/posterior wall ratio) >1.3 [32] or segmental hypertrophy confined to the LV apex [33], respectively.

CMR-FT analysis was carried out using CVi42 (Circle Cardiovascular Imaging Inc. Calgary, Canada). LV endo- and epicardial borders were manually traced at end-diastole in ECG-gated bSSFP 4- and 2-chamber long-axis sequences using a point-and-click approach. The automatic border tracking algorithm of the software was applied to track image features throughout the cardiac cycle. Tracking was visually reviewed and manually corrected by border adjustment with consecutive reapplication of the algorithm if necessary. To adjust for morphological differences between the HHD and HCM cohorts, GLS was also investigated in the previously reported “equal LVWT subgroup” that was matched for LVWT, gender, LVH, and assembled with similar age, LVMI, and T_1_ values [27].

LV LGE images were obtained using a three dimensional (3D) phase sensitive inversion-recovery (PSIR) sequence with spectral fat saturation pre-pulses during the end-diastolic phase approximately 15 minutes after administration of 0.1 mmol/kg body weight gadobenate dimeglumine (Multihance, Bracco Diagnostics Inc., Monroe Township, New Jersey, US). LGE presence and percentage (%LGE) were measured by an experienced (level 3 trained) reader (U.N.) blinded to clinical and laboratory data using an automated LV contour and LGE area quantification algorithm [34]. Accurate measurements were assured by visual review of all contours. Manual epi- and endocardial contour correction and adjustment of a gray-scale threshold to correct/define areas of visually identified LGE [35] was conducted if necessary. %LGE was calculated by summing the area of LGE in all short-axis slices, which was expressed as a volumetric proportion of the total LV myocardium.

Native myocardial T_1_ mapping was conducted using the slice-interleaved T_1_ mapping (STONE) sequence, which allows acquisition of 5 slices in the short-axis plane during a 90 seconds free breathing scan, as previously describe [36]. A 2-paramter fit model and voxel-wise curve fitting were applied to estimate individual T_1_ maps using custom software (MedIACare, Boston, Massachusetts, US). An adaptive registration of varying contrast-weighted images enabled motion correction [37]. The five resulting T_1_ maps were averaged to obtain the global native T_1_ value. Septal T_1_ and apical T_1_ were calculated as the average value of segments 2, 3, 8, 9 or 13-16/17 of the American Heart Association 16/17 segment model [38], respectively. LGE positive areas were excluded from T_1_ quantification.

### Reproducibility

CMR-FT was performed by a single investigator (U.N.). For reproducibility purposes, 20 randomly selected cases (10 HHD, 10 HCM) were reanalyzed by the same reader ≥ 4 weeks later and by a second blinded investigator (L.M.). Both were blinded to clinical information. Reproducibility of global native T_1_ and LGE extent were tested based on 50 randomly selected cases and LGE quantification available in clinical reports (n = 26), respectively.

### Statistical analysis

Data were analyzed using SPSS (version 17.0; International Business Machines Corp., Armonk, New York, USA). Normality of data distribution was determined using the Kolmogorov-Smirnov test and visual inspection of Q-Q plots. The Chi-squared test, two-sample t-test or the Mann-Whitney U-test was conducted as appropriate. Correlation between variables was tested by Pearson and Spearman ρ correlation coefficients as appropriate. Univariate and multivariate logistic regression was used to test the ability of CMR measurements to discriminate between HHD and HCM. To test for independent association of CMR measurements in the context of demographic characteristics and >1 maging marker multiple logistic regression analyses were conducted. Specificity, sensitivity, and discriminatory accuracy, cut-off values and area under the curve, were derived from receiver-operating characteristics (ROC) curve analyses using the Youden’s index. Areas under ROC curves were compared using the DeLong method. The intraclass correlation coefficient for a 2-way mixed- or random-effects model with absolute agreement was calculated to assess the intra- and interobserver reproducibility. Statistical significance was defined as a 2-sided P-value <0.05 and was Bonferroni corrected for multiple cohort comparisons.

## Results

**Fig 1:**
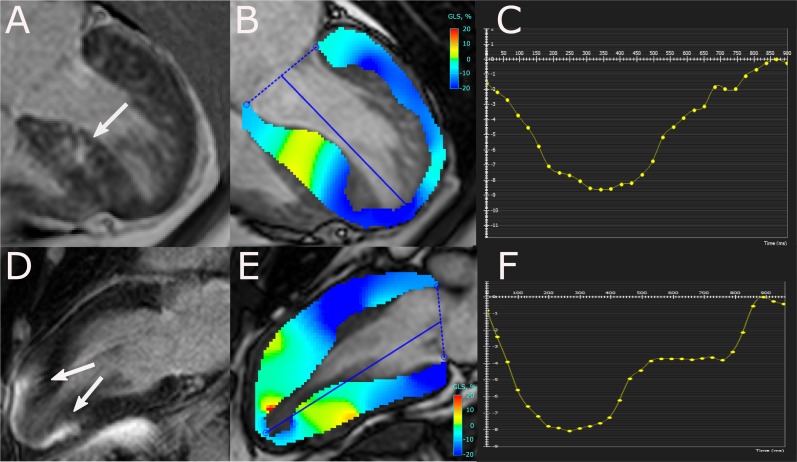
**Representative images of patients with asymmetric (A-C) and apical HCM (D-F).** Depicted are 4- (A) and 2-chamber (D) PSIR images with late gadolinium enhancement (LGE) (arrows), peak systolic longitudinal strain maps superimposed on corresponding cine-images (B, E), and resulting longitudinal strain curves. Areas of large confluent LGE (A, D) and regions with significantly attenuated strain (B, E) overlap. The patient with asymmetric HCM had an indexed left ventricular (LV) mass of 83 g/m^2^, LGE extent of 22.8%, and a GLS of -9.8%. The patient with apical HCM had an indexed LV mass of 63 g/m^2^, LGE extent of 20.3%, and a GLS of -8.9%.

The clinical characteristics are summarized in Table 1. In comparison to HCM patients, subjects with HHD were older (P = 0.012), had higher systolic and diastolic blood pressures (P<0.001 for both), and a higher body surface area (P = 0.003).The HCM cohort included patients with asymmetric septal hypertrophy (n = 62, 58%), concentric hypertrophy (n = 26, 24%), and apical variant (n = 19, 18%; Fig 1) [32]. HCM group stratification by LV hypertrophy type is summarized in S1 Table. LGE was assessed in 164 patients (34 HHD, 97 HCM, and 33 healthy controls; S2 Table.

**Table 1:** Demographic data and cohort characteristics in HHD, HCM and healthy control groups.

	HHD (n = 53)	HCM (n = 107)	Healthy Controls (n = 64)
Age, years	60±10	55±14^‡^	54±14
Sex, male n (%)	44 (83)^§^	75 (70)^§^	32 (50)
Body surface area, m^2^	2.1±0.2^§^	2.0±0.2^†^^ǁ^	1.9±0.3
Systolic Blood Pressure, mmHg	134±16^§^	127±18*	123±15
Diastolic Blood Pressure, mmHg	78±12^§^	75±10*	73±10
Heart Rate, bpm	67±11	67±9	69±11
New York Heart Association, stage			
Stage II, n (%)	9 (17)^#^	12 (11)^#^	0 (0)
Stage III, n (%)	0 (0)	3 (3)	0 (0)
Caucasian, n (%)	36 (70)	66 (62)^ǁ^	54 (84)
Hypertension, n (%)	53 (100)^§^	53 (50) *	25 (39)
Medications, n (%)	47 (87)^§^	66 (62)*^§^	20 (31)
ACEI/ARB, n (%)	31 (58)^§^	30 (28)^†^^§^	13 (20)
Beta-Blocker, n (%)	28 (53) ^§^	39 (36) ^†^^§^	7 (11)
Calcium Channel Blocker, n (%)	25 (47) ^§^	21 (20)*	10 (16)
Diuretics, n (%)	17 (32)^ǁ^	15 (14)^†^	8 (13)
Dyslipidemia, n (%)	38 (72)^ǁ^	62 (60)	30 (47)
Diabetes mellitus, n (%)	13 (25)^ǁ^	15 (14)^ǁ^	3 (5)
Serum Creatinine, mg/dl	1.1±0.3^§^	1.0±0.2^§^	0.8±0.2
Estimated Glomerular Filtration Rate, %	73±19^§^	80±20^ǁ^	92±23

ACEI, angiotensin converting enzyme inhibitor; ARB, angiotensin II receptor blocker.

* P<0.001 compared with HHD

† P<0.01 compared with HHD

‡ P<0.02 compared with HHD

§ P<0.001 compared with healthy controls

ǁ P<0.01 compared with healthy controls

# P<0.02 compared with healthy controls

P values were Bonferroni corrected (0.05/3) to account for multiple cohort comparisons.

HCM in comparison to HHD patients (Table 2) had an increased LV mass index (76±27 vs. 63±17 g/m^2^, P = 0.002), maximum LVWT (17 [15; 20] vs. 13 [12; 14] mm, P<0.001), LGE volume (0.3 [0; 3.4] vs. 0 [0; 0.1] ml, P<0.001) and global native T_1_ (1097±36 vs. 1066±36 ms, P<0.001). These differences remained significant after adjustment for age and body surface (S3 Table). There was no significant difference regarding LVEF and LV volumes between both cohorts.

**Table 2:** Cardiovascular magnetic resonance parameters in HHD, HCM and control groups.

	HHD (n = 53)	HCM (n = 107)	Healthy Controls (n = 64)
Cardiac Volumes/Diameters			
LV end-diastolic volume index, mL/m^2^	73±14	74±13	72±18
LV end-systolic volume index, mL/m^2^	29±10	26±8	28±7
RV end-diastolic volume index, mL/m^2^	71±16	66±13^§^	77±15
RV end-systolic volume index, mL/m^2^	28±11	24±8^†^^§^	32±9
LV function			
LV ejection fraction, %	63±8	65±6^§^	62±5
Global longitudinal strain, %	-16.5±3.3	-14.7±3.8^†^^§^	-17.2±2.0
4 Chamber longitudinal strain, %	-16.3±3.3	-14.3±4.0^†^^§^	-17.0±2.0
2 Chamber longitudinal strain, %	-16.6±3.6	-15.1±4.2^§^	-17.3±2.7
LV hypertrophy, n (%)	11 (21)^§^	48 (45)^†^^§^	0 (0)
LV mass index, g/m^2^	63±17^§^	76±27^†^^§^	45±11
LV anteroseptal wall thickness, mm	12 [12;13]^§^	15 [11;17]*^§^	8 [7;9]
LV inferoseptal wall thickness, mm	9 [8;12]^§^	9 [8;11]^§^	7 [6;7]
LV wall asymmetry, n (%)[32]	13 (25)	55 (51)^†^^§^	9 (14)
Maximum LVWT, mm	13 [12;14]^§^	17 [15;20]*^§^	8 [7;10]
Fibrosis markers			
LGE, n (%)	9 (26)^§^	57 (58)^†^^§^	0 (0)
LGE volume, ml	0 [0;0.1]^§^	0.3 [0;3.4]*^§^	0 [0;0]
Percent LGE, %	0 [0;0.1]^§^	0.2 [0;1.8] ^†^^§^	0 [0;0]
Global native T_1_, ms	1073±25	1097±36*^ǁ^	1079±32
Septal native T_1_, ms	1066±36	1099±41*^§^	1070±35

LGE, late gadolinium enhancement; LV, left ventricular; LVWT, LV wall thickness; RV, right ventricular.

* P<0.001 compared with HHD

† P<0.01 compared with HHD

‡ P<0.02 compared with HHD

§ P<0.001 compared with healthy controls

ǁ P<0.01 compared with healthy controls

# P<0.02 compared with healthy controls

P values were Bonferroni corrected (0.05/3) to account for multiple cohort comparisons.

### Group comparison for global longitudinal strain

**Fig 2:**
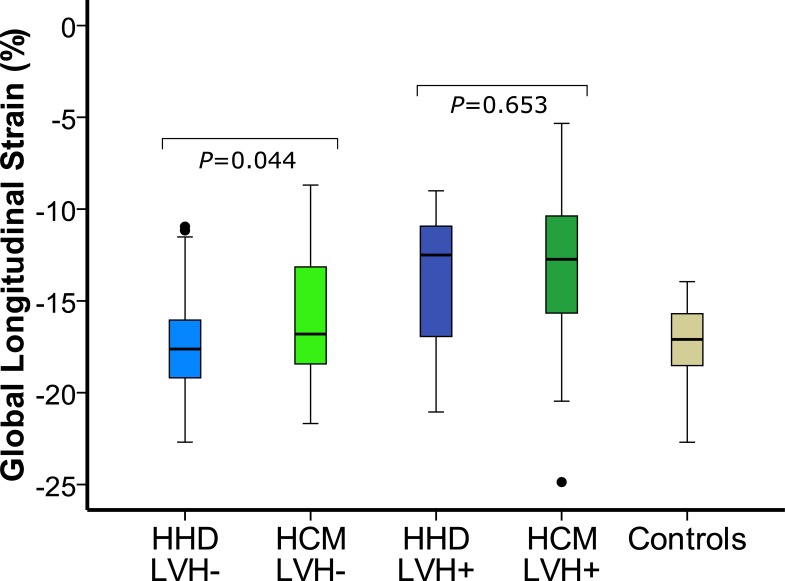
Presence of left ventricular hypertrophy (LVH) and discrimination between hypertensive heart disease (HHD) and hypertrophic cardiomyopathy (HCM). Box plots for cardiovascular magnetic resonance myocardial feature tracking (CMR-FT) global longitudinal strain (GLS) in HHD (blue), HCM (green), and controls (beige). Groups were split according to presence (LVH+, dark color) or absence (LVH-, light color) of left ventricular hypertrophy (LVH) defined according to the gender specific cut-off of LV mass index [29]. Illustrated is the influence of LVH on disease discrimination: GLS differentiates between HHD and HCM in LVH- patients, whilst it does not in LVH+ patients.

Global longitudinal strain (GLS) was significantly higher in HCM compared to HHD patients (Table 2; P = 0.004). This difference remained significant after adjustment for age and body surface area (S3 Table). The HHD to HCM differentiation was predominantly supported by patients with milder disease, as GLS was significantly different in subgroup analysis of patients with increased LVWT but without the LV mass index beyond the gender specific cut-off for LVH [29] (HHD_LVH-_ vs. HCM_LVH-_, GLS: -17.2±2.8 vs. -15.9±3.3%, P = 0.044), whilst it was not in the subgroup with LVH (HHD_LVH+_ vs. HCM_LVH+_, GLS (%):-13.8±3.8 vs. -13.2±3.8%, P = 0.653; Fig 2). We observed a difference in GLS between controls and HCM, HCM_LVH-_ or HHD_LVH+_ patients (P<0.001, P = 0.017, and P = 0.015, respectively). Comparisons of LV mass index in each subgroup showed no significant difference between HHD and HCM (HHD_LVH-_ vs. HCM_LVH-_, LV mass index: 56.8±11.4 vs. 58.4±12.9 g/m^2^, P = 0.503; HHD_LVH+_ vs. HCM_LVH+_, LV mass index: 89.0 [87.0; 92.0] vs. 91.8 [82.4; 105.8] g/m^2^, P = 0.414). The GLS difference between LVH+ and LVH- patients was significant for both disease (HHD, P = 0.015; HCM, P<0.001). GLS was significantly higher in the LGE positive HHD cohort (-13.8±3.8 vs. -17.2±2.8, P = 0.015), as well as the LGE positive HCM cohort (-13.4±3.6 vs. -16.2±3.6%, P<0.001). The maximum LVWT and prevalence of LVH were significantly greater in HHD and HCM patients in whom LGE was detected by CMR (P<0.05). GLS was different between concentric (-16.4±4.1%) and apical HCM (-13.3±3.9%, P = 0.015), but not in the comparison of asymmetric (-14.4±3.4%) with concentric or apical HCM after adjustment for multiple cohort comparison (P = 0.042 and P = 0.255, respectively). In the “equal LV wall thickness subgroup” [27] GLS did not differentiate between patients with HHD and HCM (P = 0.172, S4 Table).

### Relationship analysis of global longitudinal strain

**Fig 3:**
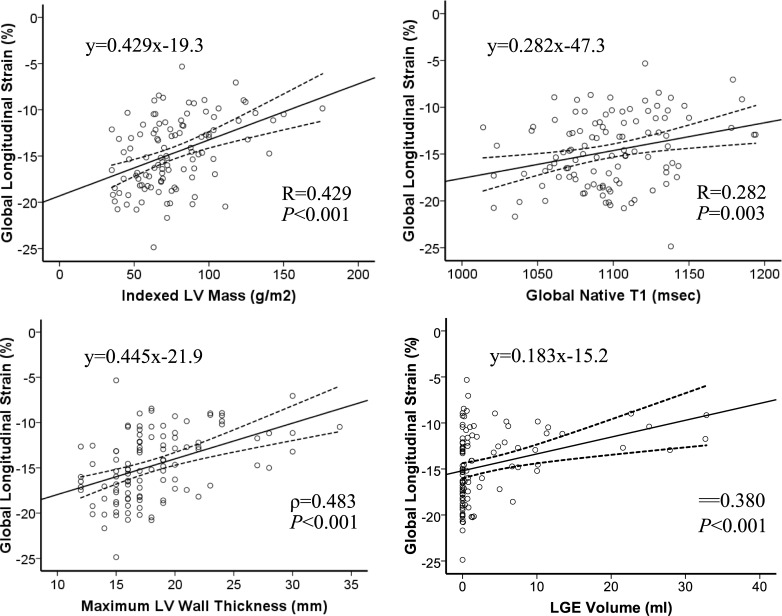
Associations of GLS, LV hypertrophy and fibrosis. Scatter plots for CMR-FT GLS and variables related to the extent of left ventricular hypertrophy and myocardial fibrosis in HCM patients. Lines indicate the best-fit line and 95% confidence interval for the mean.

In HCM patients, GLS was associated with LV end-diastolic volume, LV ejection fraction, LV hypertrophy markers, global native myocardial T_1_, and LGE volume (P<0.05; Table 3, Fig 3). When focusing on HCM patients with LGE, the association between GLS and LGE volume was however not significant (P = 0.157). In HHD patients, GLS was only associated with LV end-diastolic volume, LV ejection fraction and LV mass index (Table 3).

**Table 3:** Relationship between GLS and CMR measurements in HHD, HCM, and control groups.

	HHD		HCM		Healthy Controls	
	R/ρ	P-value	R/ρ	P-value	R/ρ	P-value
LV end-diastolic volume index, mL/m^2^	0.182	0.193	0.152	0.117	0.329	0.008
LV end-systolic volume index, mL/m^2^	0.402	0.003	0.289	0.003	0.351	0.005
Stroke volume index, ml	-0.111	0.428	-0.038	0.700	0.082	0.525
LVMI, mg/m^2^	-0.493	<0.001	-0.329	0.001	-0.328	0.009
Maximum LVWT, mm	0.419	0.002	0.429	<0.001	0.068	0.600
Percentage of LGE, %	0.132	0.455	0.367	<0.001	-	-
LGE volume, ml	0.146	0.411	0.380	<0.001	-	-
Global native T_1_, ms	0.175	0.174	0.483	<0.001	0.137	0.285

LGE, late gadolinium enhancement; LVMI, left ventricular mass index; LVWT, left ventricular wall thickness.

Two multiple linear regression models, each including 1 variable representing extent of hypertrophy (LV mass index, maximal LVWT) and global native T_1_ (all adjusted by age and sex), revealed the independent role that extent of hypertrophy plays in the attenuation of GLS in HCM (standardized regression coefficients and P values for the 2 tested variables: 0.39, P<0.001 each) and in HHD patients (standardized regression coefficients and P values for the 2 tested variables: 0.51, P<0.001; 0.30, P = 0.032, respectively). Global native T_1_ did not contribute significantly (P>0.05) to these regression models. An exchange of global native T_1_ with LGE volume provided similar results in HCM patients (standardized regression coefficients and P values for the 2 tested variables: 0.388, P<0.001; 0.386, P<0.001, respectively), and showed a significant contribution of LGE to the regression models (standardized regression coefficient and P values of LGE volume for the each model: 0.186, P = 0.030; 0.172, P = 0.049, respectively).

### Discrimination between HHD and HCM

**Fig 4:**
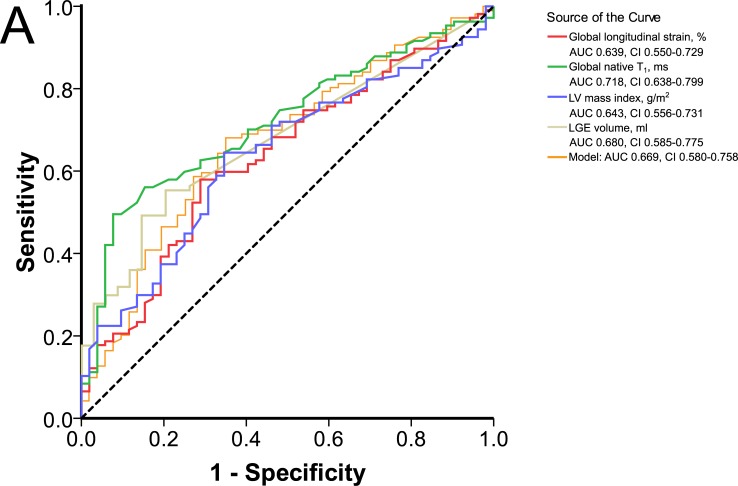
Discrimination of HHD and HCM by CMR markers. Receiver-operating characteristic curves in discrimination between HHD and HCM for single CMR markers and the multivariate regression model listed in Table 4 (GLS and LV hypertrophy (yes/no)).

In univariate analysis, GLS had diagnostic accuracies similar to hypertrophy and fibrosis markers (Table 4, Fig 4). Areas under the ROC curves comparison between GLS and global native T_1_ (P = 0.131), LGE volume (P = 0.100), or LV mass index (P = 0.967) showed no significant differences. In a multivariate binary logistic regression analysis including the prevalence of LVH and GLS, a trend for GLS to independently discriminate between HCM and HHD was observed (P = 0.066). With a sensitivity of 68% and a specificity of 64%, the model had a better test accuracy than each of its parts. In a multivariate logistic regression with forward selection of all markers represented in Table 4, only global native T_1_ (P = 0.015) and LGE prevalence (P = 0.018) remained significantly associated with disease prediction.

**Table 4:** Results of ROC and binary logistic regression analyses of CMR parameters for discrimination of HHD vs. HCM subjects.

Biomarker			Specificity (95% CI)	Sensitivity (95% CI)	PPV (95% CI)	NPV (95% CI)	Diagnostic Accuracy (95% CI)
Univariate Analysis	AUC (95% CI)	Cut-off Values					
GLS, %	0.639 (0.550–0.729)*	-15.7	72 (58–83)	58 (48–67)	81 (72–87)	46 (39–53)	63 (55–70)
Global Native T_1_, ms	0.718 (0.638–0.799)^†^	1097	92 81–98)	50 (40–59)	93 (84–97)	47 (42–52)	64 (56–71)
LGE (present)	0.656 (0.551–0.760)*	…	74 (56–87)	58 (47–67)	86 (80–92)	37 (30–45)	61 (53–70)
LGE volume, ml	0.680 (0.585–0.775)*	0.15	79 (62–91)	56 (45–66)	89 (80–94)	39 (32–45)	62 (53–70)
LV hypertrophy (present)	0.621 (0.531–0.710)*	81 (♂), 61 (♀)[29]	79 (66–89)	45 (35–55)	81 (71–88)	42 (36–47)	56 (48–64)
LV mass index, g/m^2^	0.643 (0.556–0.731)*	65.2	66 (52–78)	64 (55–74)	79 (72–85)	48 (40–56)	65 (57–72)
Multivariate analysis							
	Wald	Exp(B) (95% CI)					
GLS, %	3.380	1.102 (0.994–1.223)	64 (50–77)	68 (59–77)	79 (72–85)	50 (42–58)	67 (59–74)
LV hypertrophy (y/n)	4.227	0.424 (0.187–0.961)*					

For the multivariate model: χ^2^: 12.7, P = 0.002; -2Log LH:190.5, Cox & Snell R^2^:0.077, Nagelkerke R^2^: 0.107. Youden’s indexes for GLS, global native T1, LGE volume, LV mass index and the multivariable analysis were 0.296, 0.418, 0.351, 0.305 and 0.324, respectively. Gender specific cut-off values for LV mass index were used to define LV hypertrophy [29]. AUC, area under the curve; CI, confidence interval; GLS, global longitudinal strain; HCM, hypertrophic cardiomyopathy; LGE, late gadolinium enhancement; LH likelihood; LV, left ventricle; LVEF, LV ejection fraction; LVWT, LV wall thickness; NPV, negative predictive value; PPV, positive predictive value; ROC, receiver operating characteristics

*P<0.05

^†^P<0.001.

### CMR measurement reproducibility

**Fig 5:**
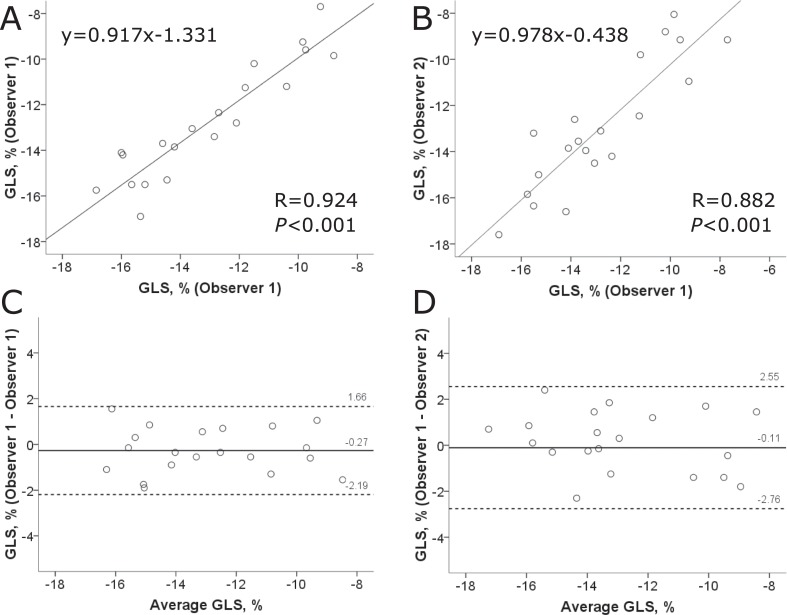
CMR-FT GLS reproducibility. Linear Regression and Bland-Altman plots illustrating intra- (A, C) and interobserver variability (B, D) of global longitudinal strain (GLS) measurements in a subset of randomly selected patients.

The intra- (ICC 0.96, 95% CI 0.90–0.98) and interobserver agreement (ICC 0.94, 95% CI 0.84–0.98) for CMR-FT GLS measurements was excellent. Bland-Altman analyses showed narrow limits of agreement for GLS on intra- and interobserver level (Fig 5). The inter-observer agreement for LGE quantification (ICC 0.88, 95% CI 0.58–0.96) and global native T_1_ (ICC 0.95, 95% CI 0.90–0.97) was excellent as well.

## Discussion

Our work investigated the role of CMR-FT in the assessment of patients with increased LVWT and showed that GLS differentiates between two of the most prevalent disease with this phenotype, HHD and HCM. This finding is consistent with publications employing STE strain assessment [10–12]. Considering CMR’s advantages over echocardiography (i.e. no interference from adjacent bone or air, decreased operator dependency) and as CMR-FT could be CMR’s answer to STE for acquisition of global strain information [39], our study confirms its relevance for the increased LVWT phenotype.

In subgroup analysis, the distinction between HHD and HCM was limited to patients with increased LVWT in the absence of LVH. Difficulties to differentiate diseases by CMR-FT GLS in the presence of LVH have been reported by Rodrigues et al., where HHD patients had a 17% higher LV mass index when compared to HCM patients [40]. In hypertensive patients GLS is more abnormal in the presence of LVH and correlates with LV mass index [8,41] indicating that contractility decline worsens with disease progression. In HCM, GLS decline occurs already prior to phenotype development [14,42]. Our finding that GLS is able to differentiate between cohorts with similar LV mass index in the absence of LVH may therefore reflect on a relative delay in GLS attenuation when comparing HHD to HCM disease progression. Related, GLS of healthy control subjects was significantly different from HCM_LVH-_, and HHD_LVH+_ patients, but similar to HHD_LVH-_ patients.

### Relationship between longitudinal strain and fibrosis

Our findings demonstrate that hypertrophy has a larger impact on CMR-FT GLS than diffuse and replacement fibrosis, as measured by native T_1_ and LGE, respectively. These findings were consistent with published STE and CMR-FT data [7,23]. Interestingly, the visualized overlap between large areas of LGE and myocardial regions with significantly attenuated strain suggests that replacement fibrosis impacts on regional deformation in HCM patients [7,43]. Related LGE was present in HHD and HCM patients with impaired longitudinal contractility. As previously shown by others [44–46], GLS correlated also with LGE extent in our HCM cohort. The investigation of LGE positive cases in isolation however showed no relationship. An artificial association between GLS and LGE extent created by the inclusion of LGE- cases is therefore possible. Yet, the small number of HCM cases with large LGE percentage (%LGE >10%, n = 6) might have contributed to this observation. Diffuse fibrosis correlated in HCM patients with GLS suggesting its contribution to longitudinal contractility decline [23]. In HHD patients fibrosis markers and longitudinal strain were not linked, which may reflect the weaker association between hypertrophy and fibrosis in hypertensive patients [22].

### Discrimination between HHD and HCM

We provide evidence for CMR-FT GLS’s capacity to differentiate between HHD and HCM. Conclusions regarding the magnitude of its diagnostic capacity require consideration of other cardiac imaging markers. For instance, both diseases have focal LGE [22,47–50], increased native T_1_ [4,22,47]_,_ diastolic dysfunction [22], as well as LVH [18,49] in common. Taking potential co-occurrences of hypertension and HCM into account, the diagnosis based on cardiac imaging alone remains often uncertain [32].

Focal fibrosis has been detected in both disease [22,47–50]. Most studies report differences for the comparison of HCM and HHD patients [22,48,49]. Likewise our data showed that LGE is more extensive and prevalent in HCM patients. Linked to the quantity of smaller LGE quantities (i.e. <1%LGE, n = 24 and n = 6 in HCM and HHD patients, respectively), LGE volume and prevalence provide similar test accuracies in our cohort. However LGE quantification may provide the stronger diagnostic marker [22,48,49]. For instance, Rudolph et al. show that %LGE but not LGE prevalence significantly differentiated between HHD and HCM patients [48]. Also the larger range of %LGE values in HCM [35] could facilitate its discriminatory ability.

Comparable to GLS [10–12] and LGE [48], the clinical application of myocardial native T_1_ mapping is limited by the overlapping data distribution between patients with different cardiac pathologies [47,51]. In HCM patients global native T_1_ is associated with LV mass index and LVWT [22,52]. In our cohort the average LV mass index difference between HHD and HCM was only 13 g/m^2^. Thus, relatively small phenotypic differences influenced probably our results. Furthermore, myocardial native T_1_ values and LVWT are correlated on segmental level [52]. By inclusion of larger quantities of non-hypertrophied segments, our whole heart coverage resulted in relatively low native T_1_ values despite regional hypertrophy. The diagnostic capacity of myocardial native T_1_ in our study differed from data reported by Hinojar et al. [22]. Next to phenotype severity and heterogeneity, differences in T1 mapping sequences probably contributed to this observation. As shown by Child et al. [53], T_1_ mapping sequences differ in their bioequivalence for discrimination between different cohorts. Therefore the use of different T_1_ mapping sequences, such as the modified Look-Locker imaging (MOLLI) sequence [53] or the slice-interleaved T_1_ mapping (STONE) sequence [36], could result in altered test accuracies.

Several studies reported differentiation between HHD and HCM by STE longitudinal strain [10–13]. Also longitudinal strain, defined as systolic shortening of the LV walls relative to its diastolic length on CMR cine images, was successfully applied by Puntmann et al. [49]. Data on CMR-FT strain is limited to the publication by Rodrigues et al. [40]. The authors reported that longitudinal strain is unable to differentiate between HHD and HCM patients with maximal LVWT ≥15 mm. These STE and CMR studies as well as our results have an overlapping data distribution in common. CMR-FT GLS contributes therefore similarly to the diagnostic process as myocardial tissue characterization and LV hypertrophy assessment, a finding that highlights the diagnostic challenge presented by patients with increased LVWT. Novel CMR imaging approaches, such as radiomic analyses of quantitative CMR images [27] or diffusion tensor CMR [54], have the potential to improve the diagnostic accuracy of cardiac imaging. In opposite to CMR-FT and established CMR tissue characterization techniques, these novel approaches are however not widely available.

### Limitations

Our study has several limitations. To adjust for the diagnostic criteria of HCM, LVWT ≥15 mm rather than LV hypertrophy [3], we defined HHD based on presence of increased LVWT [30] instead of LVH [1]. Secondly, our analyses were based on GLS assessment only, whilst other parameters of myocardial mechanics might provide additional clinical relevant information. Although large size studies are required to address an incremental value of GLS in addition to established morphological CMR markers, our study suggests a lack of such. Furthermore, functional parameters, such as LV outflow tract gradients and diastolic dysfunction [32], were not considered. Finally, our findings are based on a small single center cohort using a 1.5T CMR scanner and single CMR-FT software, whilst larger multicenter, multivendor studies are required to validate our results for widespread clinical application.

### Conclusions

Our study demonstrated that CMR-FT GLS differentiates HHD from HCM. This distinction is mostly observed in patients with increased LVWT and absence of LVH. GLS’s diagnostic accuracy is similar to CMR markers of myocardial fibrosis and LVH. However GLS’s discriminatory ability limits its clinical application and emphasizes the difficulty to differentiate the two diseases based on cardiac imaging alone.

## Supporting information

S1 Table Demographic data and cohort characteristics in subjects with late gadolinium enhancement. LV, left ventricular; LVWT, LV wall thickness. * P<0.001 compared with HHD; † P<0.01 compared with HHD; ‡ P<0.02 compared with HHD; § P<0.001 compared with healthy controls; ǁ P<0.01 compared with healthy controls; # P<0.02 compared with healthy controls. P values were Bonferroni corrected (0.05/3) to account for multiple cohort comparisons.
(DOCX)

S2 Table Logistic regression models for discrimination of HHD and HCM. GLS, global longitudinal strain; LGE, late gadolinium enhancement; LV, left ventricle; LVWT, LV wall thickness.
(DOCX)

S3 Table Cardiovascular magnetic resonance parameters in the HCM group according to LV hypertrophy type. LGE, late gadolinium enhancement; LV, left ventricular; RV, right ventricular. * P<0.001 compared with asymmetric HCM; † P<0.02 compared with asymmetric HCM; ‡ P<0.001 compared with concentric HCM; § P<0.02 compared with concentric HCM. P values were Bonferroni corrected (0.05/3) to account for multiple cohort comparisons.
(DOCX)

S4 Table CMR characteristics of the equal LVWT subgroup. HCM, hypertrophic cardiomyopathy; HHD, hypertensive heart disease; LVWT, left ventricular wall thickness. Clinical characteristics of the equal LVWT subgroup have been reported elsewhere [1].
(DOCX)

S1 Data Complete set of presented data. (XLS)

## Abbreviations

CMR: Cardiovascular magnetic resonance imaging
CMR-FT: Cardiovascular magnetic resonance myocardial feature tracking
GLS: Global longitudinal strain
HHD: Hypertensive heart disease
HCM: Hypertrophic cardiomyopathy
LGE: Late gadolinium enhancement
LV: Left ventricular
LVEF: Left ventricular ejection fraction
LVH: Left ventricular hypertrophy
LVWT: Left ventricular wall thickness
STE: Speckle-tracking echocardiography
