# Supplementary material for: Cardiovascular magnetic resonance feature tracking strain analysis for discrimination between hypertensive heart disease and hypertrophic cardiomyopathy
Source: PLoS One. 2019 Aug 21;14(8):e0221061. doi: 10.1371/journal.pone.0221061 (PMC6703851; doi:10.1371/journal.pone.0221061)
Supplement: S1 Table — LV, left ventricular; LVWT, LV wall thickness. * P<0.001 compared with HHD; † P<0.01 compared with HHD; ‡ P<0.02 compared with HHD; § P<0.001 compared with healthy controls; ǁ P<0.01 compared with healthy controls; # P<0.02 compared with healthy controls. P values were Bonferroni corrected (0.05/3) to account for multiple cohort comparisons. (DOCX) [file pone.0221061.s001.docx]

**S1** **Table** Demographic Data and Cohort Characteristics in subjects with late gadolinium enhancement

|  | HHD (n=34) | HCM (n=97) | Healthy Controls (n=33) |
| --- | --- | --- | --- |
| Age, years | 60±11 | 54±16 | 50±15† |
| Sex, male n (%) | 28 (78) | 71 (73) | 18 (55)‡ |
| Body surface area, m2 | 2.1±0.2 | 2.0±0.2# | 1.9±0.3* |
| Systolic Blood Pressure, mmHg | 142±15 | 128±16* | 126±17* |
| Diastolic Blood Pressure, mmHg | 86±12 | 76±14* | 73±12* |
| Heart Rate, bpm | 66±10 | 66±9 | 68±13 |
| Caucasian, n (%) | 21 (62) | 61 (62) | 25 (76) |
| Hypertension, n (%) | 34 (100) | 47 (48)* | 12 (36)* |
| Dyslipidemia, n (%) | 25 (74) | 55 (58) | 15 (45) |
| Diabetes mellitus, n (%) | 10 (29) | 13 (13) | 1 (3)† |
| Serum Creatinine, mg/dl | 1.05±0.23 | 0.99±0.20§ | 0.82±0.21* |
| Estimated Glomerular Filtration Rate, % | 76±19 | 80±20ǁ | 95±23† |
| Cardiovascular Magnetic Resonance | | | |
| LV ejection fraction, % | 62±9 | 65±7 | 62±5 |
| Global longitudinal strain, % | -15.7±3.5 | -14.6±3.9 | -17.2±1.8* |
| LV mass index, g/m2 | 66±18 | 78±27†§ | 48±12* |
| Maximum LVWT, mm | 13 [12; 14] | 17 [16; 20] *§ | 8 [6; 10] * |
| Global native T1, ms | 1074±29 | 1097±37*ǁ | 1077±30 |

LV, left ventricular; LVWT, LV wall thickness.

* P<0.001 compared with HHD

† P<0.01 compared with HHD

‡ P<0.02 compared with HHD

§ P<0.001 compared with healthy controls

ǁ P<0.01 compared with healthy controls

# P<0.02 compared with healthy controls

P values were Bonferroni corrected (0.05/3) to account for multiple cohort comparisons.
