# Supplementary material for: Cardiovascular magnetic resonance feature tracking strain analysis for discrimination between hypertensive heart disease and hypertrophic cardiomyopathy
Source: PLoS One. 2019 Aug 21;14(8):e0221061. doi: 10.1371/journal.pone.0221061 (PMC6703851; doi:10.1371/journal.pone.0221061)
Supplement: S2 Table — GLS, global longitudinal strain; LGE, late gadolinium enhancement; LV, left ventricle; LVWT, LV wall thickness. (DOCX) [file pone.0221061.s002.docx]

**S2** **Table** Logistic regression models for discrimination of HHD and HCM

|  | Variables | β-coefficient | P-value |
| --- | --- | --- | --- |
| Model GLS | GLS, % | 0.157 | 0.003 |
|  | Body surface area, m^2^ | -2.676 | 0.001 |
|  | Age, years | -0.050 | 0.003 |
| Model LV mass index | LV mass index, g/m^2^ | 0.026 | 0.005 |
|  | Body surface area, m^2^ | -2.810 | 0.001 |
|  | Age, years | -0.039 | 0.019 |
| Model LVWT | Maximum LVWT, mm | 0.754 | <0.001 |
|  | Body surface area, m2 | -2.716 | 0.013 |
|  | Age, years | -0.071 | 0.002 |
| Model LGE volume | LGE volume, ml | 0.403 | 0.021 |
|  | Body surface area, m^2^ | -2.548 | 0.012 |
|  | Age, years | -0.047 | 0.014 |
| Model T_1_ | Global Native T_1_, ms | 0.032 | <0.001 |
|  | Body surface area, m^2^ | -2.683 | 0.003 |
|  | Age, years | -0.071 | <0.001 |

GLS, global longitudinal strain; LGE, late gadolinium enhancement; LV, left ventricle; LVWT, LV wall thickness.
