# Supplementary material for: Cardiovascular magnetic resonance feature tracking strain analysis for discrimination between hypertensive heart disease and hypertrophic cardiomyopathy
Source: PLoS One. 2019 Aug 21;14(8):e0221061. doi: 10.1371/journal.pone.0221061 (PMC6703851; doi:10.1371/journal.pone.0221061)
Supplement: S3 Table — LGE, late gadolinium enhancement; LV, left ventricular; RV, right ventricular. * P<0.001 compared with asymmetric HCM; † P<0.02 compared with asymmetric HCM; ‡ P<0.001 compared with concentric HCM; § P<0.02 compared with concentric HCM. P values were Bonferroni corrected (0.05/3) to account for multiple cohort comparisons. (DOCX) [file pone.0221061.s003.docx]

**S3** **Table** Cardiovascular Magnetic Resonance Parameters in the HCM group according to LV hypertrophy type.

|  | Asymmetric HCM (n=62) | Symmetric HCM (n=26) | Apical HCM (n=19) |
| --- | --- | --- | --- |
| Cardiac volumes/dimensions |  |  |  |
| LV end-diastolic volume index, mL/m^2^ | 75±14 | 73±12 | 70±13 |
| LV end-systolic volume index, mL/m^2^ | 27±8 | 26±6 | 25±8 |
| RV end-diastolic volume index, mL/m^2^ | 66±13 | 68±14 | 63±11 |
| RV end-systolic volume index, mL/m^2^ | 23±8 | 25±8 | 22±8 |
| LV function |  |  |  |
| LV ejection fraction, % | 65±7 | 65±6 | 65±6 |
| Global longitudinal strain, % | -14.4±3.4 | -16.4±4.1 | -13.3±3.9^§^ |
| 4 Chamber longitudinal strain, % | -14.1±4.0 | -15.8±3.5 | -12.7±4.1^§^ |
| 2 Chamber longitudinal strain, % | -14.8±3.5 | -16.9±5.0 | -13.4±4.4 |
| Left ventricular hypertrophy, n (%) | 28 (45) | 10 (38) | 10 (52) |
| LV mass index, mg/m^2^ | 78±27 | 72±21 | 74±32 |
| LV anteroseptal wall thickness, mm | 17 [15;19] | 12 [10;15]* | 11 [11;13]^‡^* |
| LV inferoseptal wall thickness, mm | 8 [7;10] | 11 [8;12]^†^ | 8 [8;9]^§^ |
| Maximum LV wall thickness, mm | 18 [16;21] | 16 [15;17]^†^ | 16 [15;21] |
| LGE, n (%) | 33 (59) | 10 (40) | 14 (78)^§^ |
| LGE Volume, ml | 0.6 [0;4.4] | 0 [0;1.2] | 0.8 [0.1;6.7]^§^ |
| Percentage of LGE, % | 0.4 [0;2.0] | 0 [0;1.0] | 0.9 [0.1;4.4]^§^ |
| T_1_ mapping |  |  |  |
| Global native T1 (ms) | 1100±39 | 1089±36 | 1099±27 |
| Apical native T1 (ms) | 1101±39 | 1091±39 | 1114±38^§^ |
| Septal native T1 (ms) | 1105±45 | 1088±41 | 1093±26 |

LGE, late gadolinium enhancement; LV, left ventricular; RV, right ventricular.

* P<0.001 compared with asymmetric HCM

† P<0.02 compared with asymmetric HCM

‡ P<0.001 compared with concentric HCM

§ P<0.02 compared with concentric HCM

P values were Bonferroni corrected (0.05/3) to account for multiple cohort comparisons.
