# Supplementary material for: Cardiovascular magnetic resonance feature tracking strain analysis for discrimination between hypertensive heart disease and hypertrophic cardiomyopathy
Source: PLoS One. 2019 Aug 21;14(8):e0221061. doi: 10.1371/journal.pone.0221061 (PMC6703851; doi:10.1371/journal.pone.0221061)
Supplement: S4 Table — HCM, hypertrophic cardiomyopathy; HHD, hypertensive heart disease; LVWT, left ventricular wall thickness. Clinical characteristics of the equal LVWT subgroup have been reported elsewhere [1]. (DOCX) [file pone.0221061.s004.docx]

**S4** **Table** CMR characteristics of the equal LVWT subgroup.

|  | **HHD (n=23)** | **HCM (n=23)** | ***P-*value** |
| --- | --- | --- | --- |
| Global Longitudinal Strain, (%) | -15.5±3.8 | -17.1±3.5 | 0.17 |
| Global Native T_1_, ms | 1076±30 | 1083±31 | 0.45 |
| LV mass index, g/m^2^ | 74.9±18.2 | 74.3±20.7 | 0.92 |
| Maximal LVWT, mm | 14.5±2.0 | 14.3±2.1 | 0.78 |
| *Late Gadolinium Enhancement (n=36)* | | | |
| LGE (present), n (%) | 2 (14) | 8 (25) | 0.15 |
| LGE volume, ml | 0.0 [0.0; 0.0] | 0.0 [0.0, 0.6] | 0.19 |

HCM, hypertrophic cardiomyopathy; HHD, hypertensive heart disease; LVWT, left ventricular wall thickness. Clinical characteristics of the equal LVWT subgroup have been reported elsewhere [1].
